# Association between dietary inflammatory index and periodontitis in American adults: exploring the mediating role of biological aging

**DOI:** 10.3389/fnut.2025.1591830

**Published:** 2025-06-19

**Authors:** Haoyang Hua, Shencong Xu, Yifan Wang, Yuanna Zheng, Fangyue Xiang

**Affiliations:** ^1^School/Hospital of Stomatology, Zhejiang Chinese Medical University, Hangzhou, China; ^2^Urology & Nephrology Center, Department of Urology, Zhejiang Provincial People's Hospital, Hangzhou Medical College, Hangzhou, China; ^3^Ningbo Dental Hospital, Ningbo Oral Health Research Institute, Ningbo, China

**Keywords:** dietary inflammatory index, periodontitis, dietary pattern, inflammation, mediating effect

## Abstract

**Background:**

Dietary patterns are associated with inflammatory states. However, there are few reports about its relationship with periodontitis and its mechanism. This study investigated the relationship between dietary inflammatory index (DII) and periodontitis, and the mediating role of biological aging in this relationship.

**Methods:**

Data from the National Health and Nutrition Examination Survey from 2009 to 2014 were utilized, including adults aged 20 years and above. The relationship between DII and periodontitis was assessed using multivariate logistic regression analysis, and restricted cubic splines were employed to test for potential non-linear associations. Subgroup analyses were conducted to explore potential influencing factors related to DII and periodontitis. In addition, the mediating role of biological aging in dietary inflammatory indices and periodontitis was further explored.

**Results:**

A total of 10,096 participants were included in the study. The results indicated a positive correlation between DII scores and the prevalence of periodontitis. In the fully adjusted model, participants in the highest DII quartile had a 23% higher risk of periodontitis compared to those in the lowest quartile (OR = 1.23, 95% CI: 1.01, 1.48, *p* = 0.04). Subgroup analysis consistently observed a positive correlation between DII and the risk of periodontitis across all subgroups. Mediation analyses suggest some direct and indirect effects of biological aging between a pro-inflammatory diet and periodontitis.

**Conclusion:**

DII scores were positively associated with the prevalence of periodontitis in U.S. adults, suggesting that dietary patterns may have a significant impact on the prevalence of periodontitis. It also provides further insight into the mechanistic link between biological aging-mediated DII and the development of periodontitis.

## Introduction

Periodontitis is a chronic inflammation of the periodontal tissues caused by oral microorganisms. The complexity of this disease is due to the accumulation of plaque, an imbalance between the release of pro-inflammatory and anti-inflammatory cytokines, and the presence of microbial flora, leading to a loss of local homeostasis and a state of microbial dysbiosis (1–3). It not only causes gum bleeding, periodontal pocket formation, and loss of attachment, but also leads to tooth loosening and loss in the long term, and is considered the number one killer of oral health (4). According to statistics in the United States, 47% of adults over the age of 30 suffer from chronic periodontitis (5). Previous studies have shown that high intake of refined sugars, saturated fats, low consumption of antioxidants (e.g., vitamin C) and omega-3 fatty acids, and lifestyle and metabolic syndrome, can increase the risk of periodontitis (6, 7).

The Dietary Inflammatory Index (DII) is a literature-based assessment tool designed to quantify the inflammatory potential of the diet and predict individual levels of inflammatory markers (8, 9). This index integrates up to 45 different dietary components, including macronutrients and micronutrients, other dietary components, and foods, to assess the overall impact of dietary patterns on inflammatory responses (10). Specifically, a negative DII score indicates an anti-inflammatory effect, while a positive score indicates a pro-inflammatory effect, with higher scores indicating greater inflammatory potential. The DII has been widely used to assess dietary patterns associated with chronic inflammation in a variety of chronic diseases, including cancer, cardiovascular diseases, and diabetes (11, 12). However, research on the relationship between DII and periodontitis is limited. We hypothesize that a high level of DII is significantly associated with the prevalence of periodontitis.

Recent evidence suggests that biological aging may serve as a critical mediator linking dietary patterns to chronic inflammatory diseases. Accelerated biological aging, characterized by epigenetic alterations (e.g., DNA methylation age acceleration) and cellular senescence, has been shown to amplify systemic inflammation and impair tissue repair capacity - both key pathophysiological processes in periodontitis progression (13, 14). Notably, pro-inflammatory diets quantified by higher DII scores have been associated with accelerated epigenetic aging as measured by Horvath’s clock and PhenoAge algorithms (15). This dietary-induced aging phenotype may exacerbate periodontal tissue vulnerability through multiple pathways: (1) impaired neutrophil function and compromised oral mucosal barrier (16), (2) enhanced osteoclast activation via RANKL/OPG axis dysregulation (17), and (3) microbiome dysbiosis through redox imbalance (18). A recent cohort study showed a significant correlation between a Western diet and telomere shortening in white blood cells (19). The shortening of telomeres in white blood cells is a quantitative indicator of cellular senescence, suggesting that biological senescence is a credible mechanistic bridge. However, no studies have comprehensively examined the mediating role of multidimensional aging biomarkers in the DII-periodontitis association.

Therefore, the purpose of this study is to use the National Health and Nutrition Examination Survey (NHANES) database to assess the correlation between DII and periodontitis, providing a scientific basis for the prevention and early intervention strategies of periodontitis.

## Methods

### Database and survey populations

This cross-sectional study utilized data from the NHANES conducted by the Centers for Disease Control and Prevention from 2009 to 2014.^1^ The NHANES program assesses the health and nutritional status of the U. S. population through a stratified, multi-stage survey. Prior to participation in NHANES, all participants provided written informed consent approved by the National Center for Health Statistics (NCHS) Institutional Review Board.

**Figure 1:**
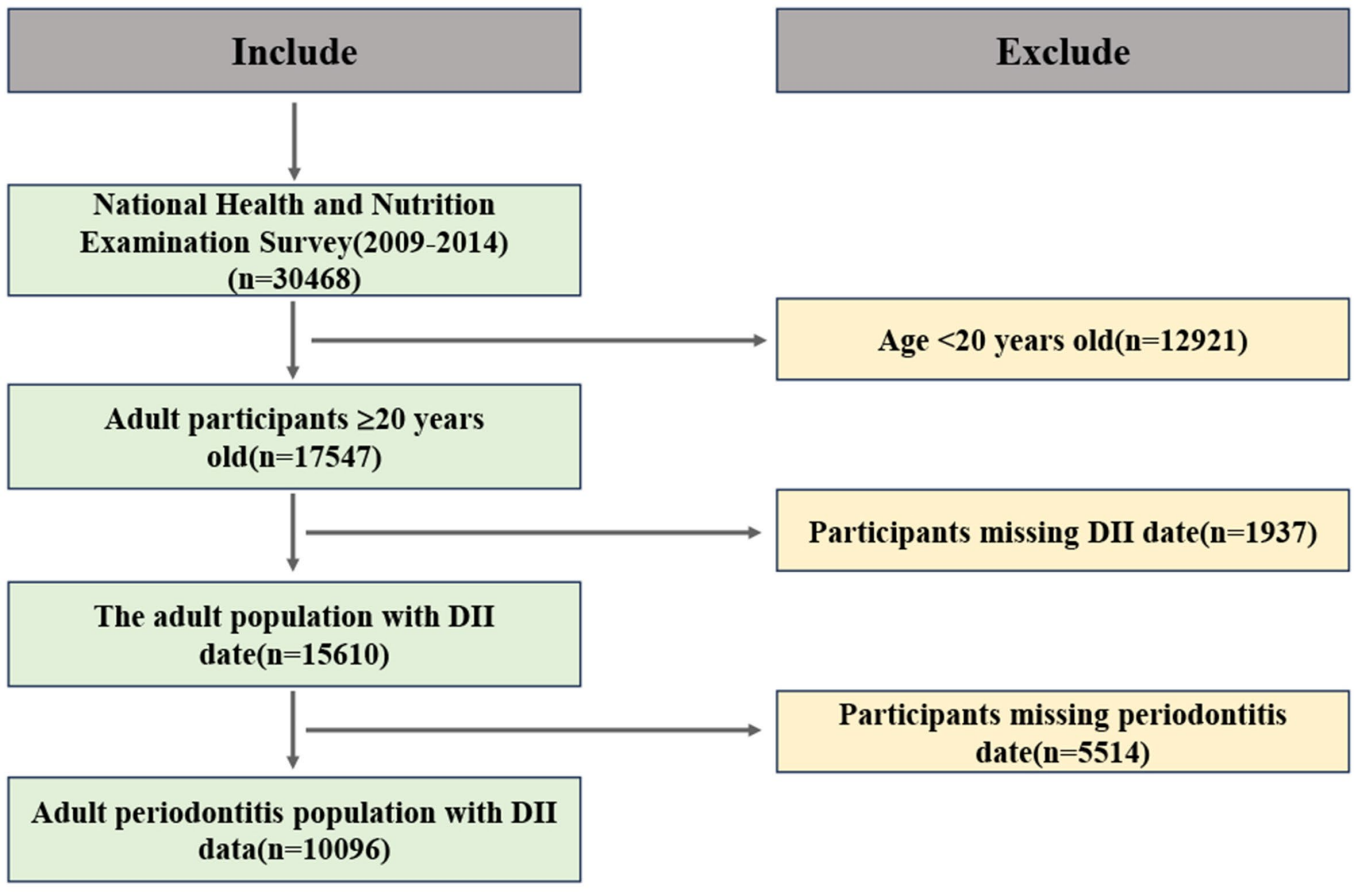
Flowchart of participant selection.

All adults aged 20 years or older with permanent teeth were eligible for a full-mouth periodontal examination. Participants in NHANES completed a survey questionnaire at home, followed by a physical examination and interview at the Mobile Examination Center. Since only a subset of NHANES participants underwent MEC examinations, we included only those who reported having a complete dental examination. Dietary quality was derived from a 24-h dietary recall and assessed using the DII score. We also included other demographic variables, such as age, gender, race, education level, marital status, smoking, alcohol consumption, and Body Mass Index (BMI). Initially, 30,468 participants were included for further analysis. Figure 1 displays a flowchart illustrating the criteria used for patient selection.

### Dietary inflammatory index

In this study, the DII was calculated as the exposure factor by recording the dietary data of participants over 2 days through a 24-h dietary recall interview conducted at the Mobile Examination Center. We calculated the average daily intake of each dietary component during the two memory days. Then, these average values were used to calculate the individual DII scores, and the same weights were given to the data of the 2 days according to the NHANES analysis guidelines. The formula for calculating the DII is as follows: DII for each nutrient or dietary component = [(Daily intake of the nutrient or dietary component - Global daily intake of the nutrient or dietary component per capita)/Standard deviation of the global daily intake of the nutrient or dietary component per capita] * Inflammatory Effect Index for the nutrient or dietary component. The sum of the DII for all nutrients or dietary components is the total DII score for the individual participant (8).

Researchers summarized the inflammatory effect scores for 45 nutrients and estimated the global average and standard deviation for each nutrient. Data on 27 of the 45 food parameters were obtainable through NHANES, including alcohol, caffeine, protein, fiber, beta-carotene, cholesterol, carbohydrates, energy, fat, n-3 fatty acids, n-6 fatty acids, polyunsaturated fatty acids, monounsaturated fatty acids, saturated fat, thiamin, magnesium, zinc, selenium, iron, riboflavin, folate, vitamin A, vitamin B-6, vitamin B-12, vitamin C, vitamin D, vitamin E, and niacin. Nevertheless, the omission of bioactive compounds like curcumin may modestly attenuate observed effect sizes, suggesting our estimates represent conservative associations. A higher score indicates a more pro-inflammatory diet, while a lower score indicates a stronger anti-inflammatory effect. The DII score was analyzed as a categorical variable, and participants were grouped into quartiles from the total sample.

### Periodontitis

A full-mouth periodontal examination was conducted by calibrated dentists to assess the periodontal status of participants. The periodontal examination included the measurement of probing depth (PD) and clinical attachment level (AL) at the Mobile Examination Center. The classification criteria for periodontal status followed the CDC/AAP case definitions: mild periodontitis (≥2 interproximal sites with AL ≥ 3 mm and ≥2 interproximal sites with PD ≥ 4 mm), moderate/severe periodontitis (≥2 interproximal sites with AL ≥ 4 mm and ≥2 interproximal sites with PD ≥ 5 mm) (4). The classification criteria for periodontal status are provided in Supplementary Table 1.

### Covariates

Socio-demographic characteristics were defined as age, gender (male/female), and race (Mexican American, other Hispanic, non-Hispanic white, non-Hispanic black, or other). Education level was assessed through a screening questionnaire and categorized as less than high school, High school or equivalent, and College or above. Marital status was classified as Married/Living with Partner, Never married, Widowed/Divorced/Separated. In the United States, the Poverty-Income Ratio (7) is defined as the ratio of a household’s self-reported income to the poverty line. Household income was divided into three levels based on PIR: low (PIR < 1.35), middle (1.35 ≤ PIR < 3.0), and high (PIR ≥ 3.0) (20). Smoking status was classified according to the following criteria: never smokers were defined as adults who had never smoked or smoked less than 100 cigarettes in their lifetime; former smokers were identified as those who reported smoking 100 or more cigarettes in their lifetime and were not currently smoking; and current smokers were defined as those who smoked 100 or more cigarettes in their lifetime and currently smoke on some days or daily. Based on these criteria, participants were divided into “Never,” “Former,” and “Current” smokers. The study categorized participants into two groups based on whether they reported excessive alcohol consumption daily. Hyperlipidemia is defined as fasting total cholesterol ≥240 mg/dL or fasting triglyceride ≥200 ng/dL (21). Hypertension was diagnosed based on four criteria, and individuals meeting any one of these criteria were classified as hypertensive. The diagnostic criteria included self-reported history of hypertension, use of antihypertensive medication, systolic blood pressure (SBP) ≥ 140 mmHg, or diastolic blood pressure (DBP) ≥ 90 mmHg (22). Individuals who reported having diabetes or taking anti-diabetic medication were considered to have diabetes.

### Statistical analysis

We employed the weights recommended by the Centers for Disease Control and Prevention (CDC), which account for sampling bias to obtain more accurate information. Continuous variables are expressed as means ± standard deviations, and categorical variables are expressed as numbers (percentages). The association between the DII and periodontitis was estimated using a multiple logistic regression model, adjusted for potential confounding factors. DII was analyzed as a categorical variable divided into quartiles. Quartile cut-points were determined using sample-weighted percentiles of the total study population (Q1: −5.28 to −0.53; Q2: −0.53 to 2.41; Q3: 2.41 to 5.47). In logistic regression models, the lowest quartile (Q1) served as the reference group. Model 1 was adjusted for gender, age, and race. Model 2 included additional adjustments for BMI, education level, marital status, Poverty-Income Ratio (7), smoking, alcohol consumption, diabetes mellitus (DM), hyperlipidemia, and hypertension. The dose–response relationship between DII and the risk of periodontitis was assessed using restricted cubic splines (RCS). Additionally, subgroup analyses were conducted to evaluate the relationship between DII and periodontitis in different subgroups.

Quasi-Bayesian causal mediation analyses were performed using the “mediator” package. These mediator models were simulated 100 times to estimate the total, direct, and indirect effects of DII on periodontitis and incorporated three biological ages [Klemera-Doubal method (KDM), PhenoAge, and homeostatic dysregulation (HD)] as potential mediators. The total effect represented the overall association between DII and periodontitis, whereas the direct effect captured the mediator-independent association. Indirect effects quantified the portion of the association mediated by a specific mediator. The proportion of mediators was calculated to assess the relative contribution of mediators to the total effect (Table 1).

**Table 1:** Baseline characteristics of the study population.

Variable	Overall	Non-Periodontitis	Periodontitis	*p-*value
Age (years)	50.62 ± 0.28	47.86 ± 0.29	54.31 ± 0.41	**< 0.0001**
Sex (%)				**< 0.0001**
Female	4,955 (51.05)	2,872 (57.98)	2083 (41.76)	
Male	4,772 (48.95)	1900 (42.02)	2,872 (58.24)	
Race (%)				**< 0.0001**
Mexican American	1,385 (8.16)	495 (5.80)	890 (11.32)	
Non-Hispanic Black	2023 (10.89)	792 (8.15)	1,231 (14.56)	
Non-Hispanic White	4,297 (68.04)	2,442 (74.04)	1855 (60.00)	
Other Hispanic	968 (5.60)	464 (5.04)	504 (6.35)	
Other Race - Including Multi-Racial	1,054 (7.31)	579 (6.98)	475 (7.77)	
BMI (%)				**0.02**
≤29.9	5,885 (62.54)	2,952 (63.91)	2,933 (60.71)	
>29.9	3,842 (37.46)	1820 (36.09)	2022 (39.29)	
Education (%)				**< 0.0001**
Less than high school	2,198 (14.86)	676 (9.03)	1,522 (22.69)	
High school or equivalent	2,113 (20.77)	856 (16.27)	1,257 (26.81)	
College or above	5,416 (64.37)	3,240 (74.70)	2,176 (50.51)	
Smoking status (%)				**< 0.0001**
Never	5,472 (56.78)	3,093 (64.79)	2,379 (46.02)	
Former	2,463 (25.83)	1,093 (24.50)	1,370 (27.60)	
Now	1792 (17.40)	586 (10.71)	1,206 (26.38)	
Marital status (%)				**< 0.0001**
Never married	1,113 (10.66)	577 (10.55)	536 (10.80)	
Married/Living with Partner	6,319 (69.13)	3,258 (72.95)	3,061 (64.00)	
Widowed/Divorced/Separated	2,295 (20.21)	937 (16.49)	1,358 (25.19)	
PIR (%)				**< 0.0001**
<1.3	3,553 (34.04)	1,568 (28.94)	1985 (40.88)	
1.3–3.5	3,336 (46.45)	2,133 (57.23)	1,203 (31.98)	
>3.5	2,838 (19.51)	1,071 (13.83)	1767 (27.13)	
Excessive drinking (%)				0.34
No	5,247 (53.72)	2,598 (54.40)	2,649 (52.81)	
Yes	4,480 (46.28)	2,174 (45.60)	2,306 (47.19)	
DM (%)				**< 0.0001**
No	1832 (13.32)	632 (9.28)	1,200 (18.75)	
Yes	7,895 (86.68)	4,140 (90.72)	3,755 (81.25)	
Hypertension (%)				**< 0.0001**
No	5,416 (60.23)	3,001 (65.82)	2,415 (52.72)	
Yes	4,311 (39.77)	1771 (34.18)	2,540 (47.28)	
Hyperlipidemia (%)				**0.01**
No	2,553 (27.01)	1,360 (28.58)	1,193 (24.90)	
Yes	7,174 (72.99)	3,412 (71.42)	3,762 (75.10)	
DII (%)				**< 0.001**
Q1	3,210 (36.67)	1,695 (39.12)	1,515 (33.38)	
Q2	3,210 (32.48)	1,560 (32.58)	1,650 (32.34)	
Q3	3,307 (30.85)	1,517 (28.29)	1790 (34.28)	

The bold value indicates *p* < 0.05.

All analyses were performed using statistical software packages R 4.4.1 and Free Statistics software version 1.8. A descriptive study was conducted for all participants. Statistical tests were two-tailed, and a *p*-value of less than 0.05 was considered statistically significant.

## Results

### Baseline characteristics

According to the diagnostic criteria provided in Supplementary Table 1, participants were divided into two groups based on periodontitis status. After excluding participants under the age of 20, a total of 17,547 individuals were included in the study. Further selection excluded participants lacking periodontitis (*n* = 5,514) and DII (*n* = 1,937) data. Ultimately, 10,096 participants were selected from the surveyed population for further analysis. The average age of participants with periodontitis was 54.3 years, which was higher than that of participants without periodontitis (47.9 years). Additionally, there was a significant intergroup difference between females (41.76%) and males (58.24%), indicating a higher risk of disease prevalence in the male population. The prevalence of periodontitis was highest among non-Hispanic whites, followed by non-Hispanic blacks. Concurrently, the prevalence of periodontitis was significantly higher among individuals with lower income levels. Data indicated that the proportion of periodontitis was higher among married or cohabitating individuals compared to those who were unmarried or divorced. Analysis of comorbidity distribution revealed significantly higher proportions of hyperlipidemia (75.10% vs. 71.42%) and diabetes (18.75% vs. 9.28%) in the periodontitis group compared to the non-periodontitis group, whereas hypertension showed an inverse pattern with lower prevalence in periodontitis patients (47.28% vs. 52.72%). Participants with periodontitis exhibited higher DII scores (Q3:34.28% vs. 28.29% in non-periodontitis group).

### The relationship between DII score and periodontitis prevalence

**Figure 2:**
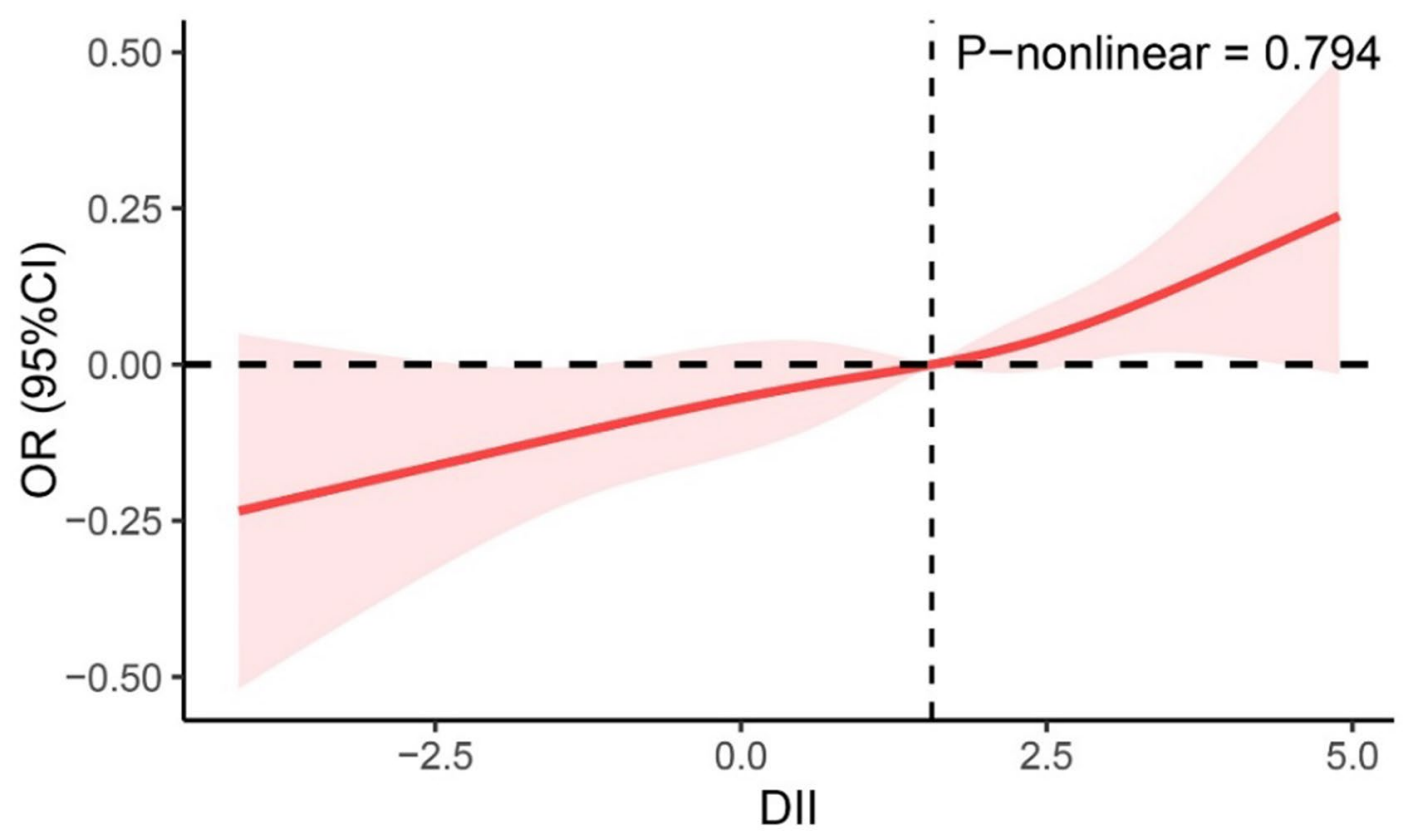
Restricted cubic spline curve between DII score and periodontitis.

From the restricted cubic spline curve in Figure 2, it can be visually understood that there is a linear relationship between the DII and periodontitis. This relationship was further explored using three multiple-regression models based on quartiles. All models demonstrated a positive correlation between DII and the prevalence of periodontitis. According to the results of the logistic regression analysis, a higher quartile of DII was associated with a higher prevalence of periodontitis. Using the lowest quartile of DII as the reference, in Model 1 (DII-Q3: OR = 1.72; 95% CI: 1.44–2.06), and Model 2 (DII-Q3: OR = 1.23; 95% CI: 1.01–1.48). The trend *p*-value for all models was less than 0.05 (Table 2).

**Table 2:** Weighted logistic regression analysis on the association between DII and periodontitis.

Exposure	Non-adjusted model		Model I		Model II	
	OR (95% CI)	*p*-value	OR (95% CI)	*p*-value	OR (95% CI)	*p*-value
DII-Q1	Ref		Ref		Ref	
DII-Q2	1.16 (0.99, 1.37)	0.07	1.25 (1.05, 1.49)	0.01	1.06 (0.90, 1.25)	0.4
DII-Q3	1.42 (1.19, 1.69)	<0.001	1.72 (1.44, 2.06)	<0.0001	1.23 (1.01, 1.48)	0.04
P for trend		<0.001		<0.0001		0.04

Non-adjusted model adjusts for: DII.

Model I adjust for: DII, Age, Race, Sex.

Model II adjust for: DII, age, race, sex, BMI, marital status, PIR, education, DM, hypertension, hyperlipidemia, excessive drinking, smoking.

DII-Q1 was the control group.

DII-Q1: −5.281-0.533, DII-Q2:0.533–2.409, DII-Q3:2.409–5.474.

### Subgroup analysis

**Figure 3:**
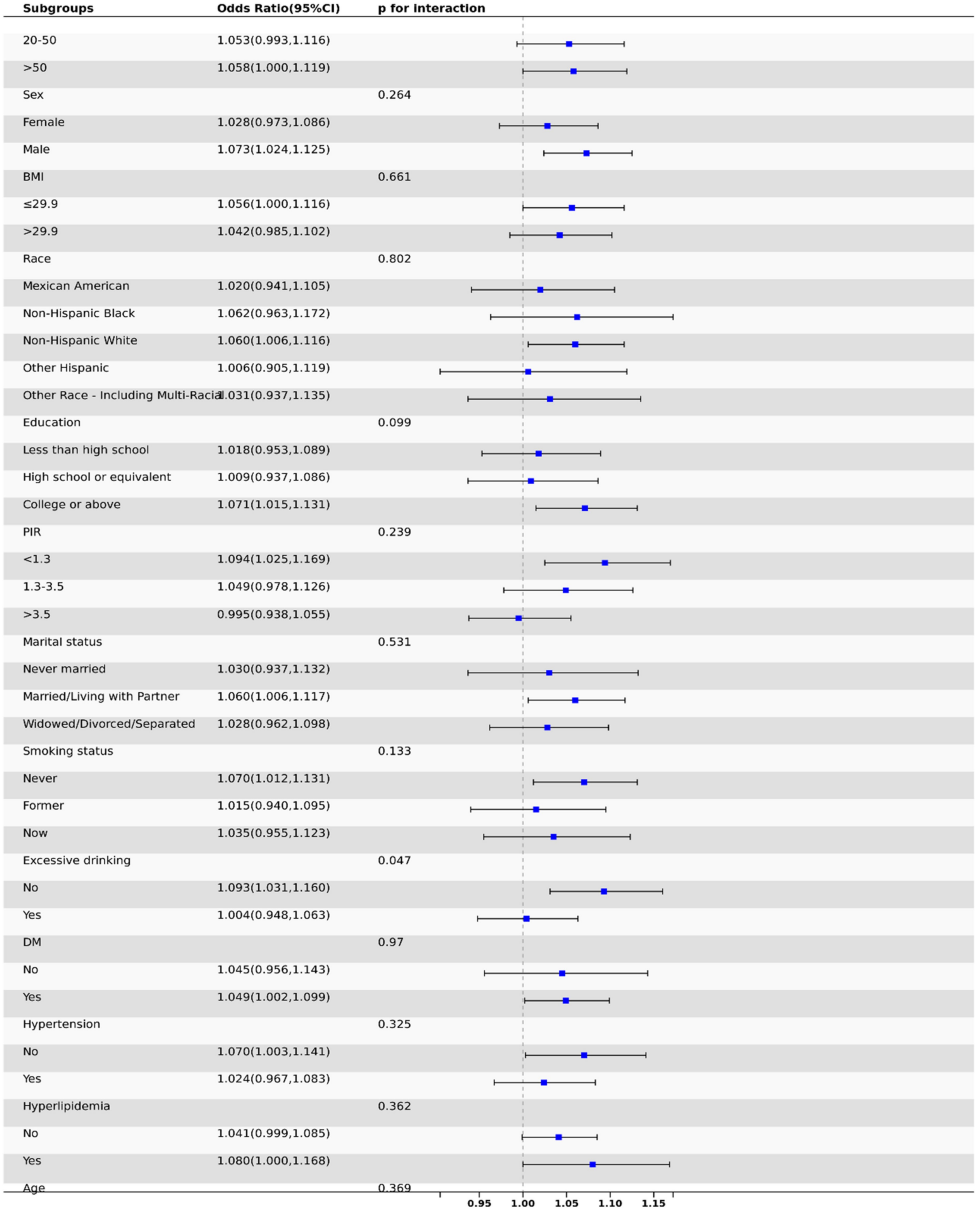
Subgroup analysis of the relationship between DII and periodontitis in forest map.

This study employed subgroup analysis to test for heterogeneity among various subgroups. After adjusting for covariates, we found no significant differences among subgroups in terms of age, gender, race, education, smoking status, marital status, BMI, diabetes, hypertension, and hyperlipidemia (*p* > 0.05 for all), indicating that the degree of association among different subgroup populations was consistent (Figure 3).

### Mediation analysis

Mediation analyses showed that DII exerted a significant indirect effect on periodontitis via biomarkers of aging after adjusting for age, sex, race, body mass index, and underlying disease (Table 3). Of these, KDM mediated only 7.1% (95% CI: 3.0–20.5%) of the total effect, but still had significant direct and indirect effects (*p* < 0.001). For HD and PhenoAge, no significant direct mediating effects were observed, but both observed significant indirect effects (*p* < 0.05).

**Table 3:** Mediation analysis of the relationship between DII and periodontitis.

Independent variable	Mediator	Total effect		Indirect effect		Direct effect		Proportion mediated, % (95% CI)
		Coefficient (95% CI)	*p* value	Coefficient (95% CI)	*p* value	Coefficient (95% CI)	*p* value	
DII	HD	0.00672 (−0.00063, 0.01516)	0.060	0.00057 (0.00011, 0.00127)	<0.001	0.00615 (−0.00109, 0.01473)	0.080	8.5 (−5.0, 61.7)
DII	KDM	0.00710 (0.00271, 0.01203)	<0.001	0.00051 (0.00026, 0.00083)	<0.001	0.00659 (0.00219, 0.01162)	<0.001	7.1 (3.0, 20.5)
DII	PhenoAge	0.00671 (−0.00084, 0.01501)	0.060	0.00087 (0.00014, 0.00168)	0.020	0.00584 (−0.00177, 0.01450)	0.120	11.3 (−19.4, 110.3)

The mediation analyses were adjusted for age, sex, race, BMI, marital status, PIR, education, DM, hypertension, hyperlipidemia, excessive drinking, and smoking.

DII, dietary inflammatory index; HD, homeostatic dysregulation; KDM, Klemera-Doubal method.

## Discussion

In this cross-sectional study, the relationship between DII scores and the incidence of periodontitis was extensively analyzed using data from American adults in the NHANES 2009–2014. The analysis of 10,096 participants concluded that DII is positively correlated with the incidence of periodontitis. Compared to the lowest quartile, the highest quartile of DII significantly increased the incidence of periodontitis. After adjusting for a range of potential confounding factors, the conclusion remained valid and was found to be stable in various validations.

An increasing body of research is revealing a close link between dietary habits and the development of periodontitis (23, 24). Specifically, a randomized controlled trial found that adopting an anti-inflammatory, plant-based diet rich in omega-3 fatty acids, vitamin C, vitamin D, and dietary fiber significantly reduced the incidence of gingivitis in individuals following a Western dietary pattern (25, 26). In an experimental study targeting gingivitis, participants experienced a marked improvement in gum bleeding after 1 month of dietary adjustments that emphasized reduced intake of processed carbohydrates and refined sugars (27). Additionally, diet-induced dysbiosis of the oral microbiome may exacerbate oxidative stress and neutrophil hyperreactivity. High intake of saturated fats and refined carbohydrates has been shown to increase the abundance of periodontal pathogens (e.g., *Porphyromonas gingivalis*) while suppressing commensal species (*Streptococcus salivarius*). This dysbiosis disrupts oral biofilm homeostasis, promoting sustained inflammation through Toll-like receptor activation and impaired resolution pathways. Furthermore, high-fat diets (HFDs) are considered pro-inflammatory, leading to increased levels of circulating free fatty acids (FFAs) in the bloodstream (28). The elevation of FFAs can activate immune cells, including macrophages and T-lymphocytes, thereby triggering the release of pro-inflammatory cytokines and promoting the development of local inflammation (29). Hyperlipidemia induced by HFDs has been shown to stimulate inflammatory signaling pathways, such as toll-like receptor 4 (TLR4) and nuclear factor kappa-B (NF-κB). The activation of these pathways promotes the transcription of inflammatory genes. In the context of this study, a significant association was observed between the prevalence of periodontitis and hyperlipidemia. These findings support the correlation between pro-inflammatory diets and periodontitis.

An increasing body of evidence suggests that dietary patterns may be closely associated with inflammatory biomarkers. Roberta et al. found that individuals with a long-term pro-inflammatory diet have higher concentrations of interleukin-33 (IL-33) and macrophage chemotactic protein 1 (MCP-1) (30). IL-33 is a member of the IL-1 family and plays an important role in inducing and regulating immune responses (31). A previous study found that IL-33 can induce the production of various cytokines, including MCP-1. MCP-1 was significantly increased in periodontitis patients and was associated with bleeding on probing gums (32). IL-33 and MCP-1 can interact at the initial stage of inflammation, participating in the pathophysiological process of inflammatory diseases involving the activation of leukocytes (33). Corley et al. used several different inflammatory markers, including C-reactive protein (CRP), IL-1, IL-2, IL-6, interferon-gamma (IFN-*γ*), and tumor necrosis factor-alpha (TNF-*α*), to verify the changes in serum inflammatory markers in periodontitis (34). These findings suggest that periodontal health may be influenced by dietary inflammation through systemic immune modulation. Therefore, different diets may alter the cytokine homeostasis in the gingival environment and promote the initial changes in local tissue inflammation. Dental professionals should incorporate dietary counseling into periodontal management with targeted anti-inflammatory therapy (35). Public health policies could leverage DII screening to identify high-risk populations for targeted nutrition education.

Emerging evidence suggests biological aging may mediate the diet-periodontitis link through inflammatory pathways. Pro-inflammatory diets have been shown to accelerate epigenetic aging biomarkers such as DNA methylation age (measured by GrimAge acceleration), while shortened telomere length and elevated epigenetic age are strongly associated with periodontal destruction in cross-sectional population studies (36–39). Mechanistically, inflammatory cytokines (e.g., IL-6, TNF-*α*) activated by DII promote cellular senescence through STAT3-mediated pathways and mitochondrial dysfunction (40). This creates a potential mediation loop where diet-induced inflammation drives biological aging, which subsequently increases periodontal tissue vulnerability. Our findings extend prior mechanistic understanding by proposing biological aging as a novel mediator. The observed DII-periodontitis association may be partially explained through three aging-related pathways: (1) NF-κB-mediated epigenetic aging: Chronic inflammation induces DNA methylation changes via NF-κB activation, as evidenced by accelerated GrimAge in periodontitis patients with systemic inflammation (36, 38, 41). (2) Oxidative stress and telomere attrition: Pro-inflammatory diets exacerbate oxidative damage in periodontal fibroblasts, leading to telomere shortening observed in aging-related periodontitis cohorts (39, 42, 43). (3) Gut-microbiota-inflammation axis: Diet-modulated dysbiosis promotes systemic “inflammaging” through elevated IL-1β and TNF-*α* levels, a pathway validated in mediation analyses of aging populations (44, 45). The mediation analysis in this study revealed statistically significant yet modest mediating effects of biological aging biomarkers, with KDM accounting for 7.1% of the total effect. While the proportion mediated appears limited, these findings provide mechanistic plausibility to the hypothesis that accelerated aging phenotypes exacerbate periodontal tissue vulnerability through inflammatory amplification and impaired repair capacity.

The strength of this study lies in its data source, the NHANES, whose comprehensiveness allows for an extensive assessment of various potential confounding factors, thereby enhancing the reliability of the study’s results. Secondly, to avoid the reduced statistical power and bias that might result from directly excluding missing data, we employed multiple imputation methods to estimate the missing values. Furthermore, the study included many participants and utilized weighted analysis methods for a representative sample of the U. S. population, further enhancing the generalizability and applicability of the research findings.

Despite the study’s several strengths, it is necessary to acknowledge some limitations. First, the cross-sectional design of the NHANES data does not allow for the determination of a causal relationship between DII and periodontitis. Second, relying on two non-consecutive 24-h dietary recalls in NHANES may not fully capture habitual dietary patterns, and self-reported data are prone to measurement errors. Although NHANES employs standardized protocols to minimize recall bias, the absence of longitudinal dietary data limits causal inference. Because of the observational design, interference from unmeasured factors, including genetic predisposition, stress, and medications, cannot be ruled out. Future research should prioritize randomized trials that test the effects of anti-inflammatory dietary interventions on periodontal disease outcomes and explore longitudinal changes in aging biomarkers. Prospective studies with larger sample sizes should also be conducted to further clarify the causal relationship between DII and periodontitis.

## Conclusion

This study employed a cross-sectional research methodology, utilizing data from the NHANES database between 2009 and 2014. After applying the inclusion and exclusion criteria, a final sample of 10,096 participants was included. By employing multivariable logistic regression, subgroup analysis, and restricted cubic splines, a significant positive correlation was found between DII scores and the prevalence of periodontitis, further indicating that increased intake of pro-inflammatory diets may elevate the incidence of periodontitis. In addition, the potential role of biological senescence in the relationship between a pro-inflammatory diet and periodontitis was explored through mediation analyses.

## Data Availability

The datasets presented in this study can be found in online repositories. The names of the repository/repositories and accession number(s) can be found in the article/Supplementary material.

## Footnotes

^1^ https://www.cdc.gov/nchs/NHANES/index.htm
