# Supplementary material for: Association between dietary inflammatory index and periodontitis in American adults: exploring the mediating role of biological aging
Source: Front Nutr. 2025 Jun 19;12:1591830. doi: 10.3389/fnut.2025.1591830 (PMC12222260; doi:10.3389/fnut.2025.1591830)
Supplement: Supplementary file 1 [file Table_1.docx]

Supplementary Table 1. Periodontitis classification criteria.

| **Classification** | **Standard** |
| --- | --- |
| Mild periodontitis | ≥2 interproximal sites with AL ≥3 mm, and ≥2 interproximal sites with PD ≥4 mm (not on same tooth) or one site with PD ≥5 mm |
| Moderate periodontitis | ≥2 interproximal sites with AL ≥4 mm (not on same tooth), or ≥2 interproximal sites with PD ≥5 mm (not on same tooth) |
| Severe periodontitis | ≥2 interproximal sites with AL ≥6 mm (not on same tooth) and ≥1 interproximal site with PD ≥5 mm |
| Non-periodontitis | No evidence of mild, moderate, or severe periodontitis |
